# Prone Positioning During ECPELLA Support for Cardiogenic Shock: A Single-Center Retrospective Study

**DOI:** 10.3390/jcm15103626

**Published:** 2026-05-09

**Authors:** Hiroki Kajiura, Akinori Sawamura, Toshio Taniguchi, Keisuke Nishio, Ryuzo Imaeda, Ryu Tanahashi, Ryota Yamauchi, Hiroshi Tashiro, Norio Umemoto, Hisaaki Ishiguro, Kiyokazu Shimizu

**Affiliations:** Department of Cardiology, Ichinomiya Municipal Hospital, Ichinomiya 4918558, Aichi, Japan; a.sawamu@gmail.com (A.S.); toshio1975114@yahoo.co.jp (T.T.); nsowesto@gmail.com (K.N.); r.imaeda1993@gmail.com (R.I.); ryu.t.0170d@gmail.com (R.T.); ryouta2577@gmail.com (R.Y.); hiroshi.t0722@gmail.com (H.T.); goog1e_ab1ation@yahoo.co.jp (N.U.); sak@xc4.so-net.ne.jp (H.I.); shi03549@yahoo.co.jp (K.S.)

**Keywords:** ECPELLA, prone positioning, cardiogenic shock, venoarterial extracorporeal membrane oxygenation, Impella, mechanical circulatory support, hypoxemia, atelectasis

## Abstract

**Background**: Prone positioning improves oxygenation in acute respiratory distress syndrome, but its feasibility and safety in patients with cardiogenic shock supported by combined venoarterial extracorporeal membrane oxygenation and percutaneous transvalvular microaxial flow pump (ECPELLA) remain unclear. **Methods**: We retrospectively reviewed 65 consecutive patients receiving ECPELLA between February 2020 and August 2024. Fifteen patients underwent prone positioning for hypoxemic respiratory failure, in which dorsal atelectasis was considered a major contributing factor. Respiratory parameters, including PaO_2_/FiO_2_ ratio and dynamic lung compliance, as well as hemodynamic variables, were assessed before and after prone positioning. **Results**: Prone positioning was initiated a median of 2 days after ECPELLA initiation, with a median cumulative duration of 32 h. PaO_2_/FiO_2_ improved from 145 (IQR, 80–177) to 308 (IQR, 260–375; *p* < 0.0001). Cdyn increased modestly from 33 (IQR, 28–37) to 35 (IQR, 33–50) mL/cmH_2_O (*p* = 0.03). Hemodynamic parameters remained stable. One patient (6.7%) experienced bleeding at the ECMO cannulation site, controlled with a purse-string suture. Pressure ulcers occurred in four patients (stage 1–2) and were managed conservatively. **Conclusions**: Prone positioning during ECPELLA support was feasible and associated with improved oxygenation without significant hemodynamic compromise. Observed complications were manageable. These findings suggest prone positioning may serve as a supportive respiratory strategy in selected cardiogenic shock patients on ECPELLA. Prospective multicenter studies are warranted to confirm these results.

## 1. Introduction

Cardiogenic shock is increasingly managed with various forms of mechanical circulatory support (MCS). Among them, the combined use of venoarterial extracorporeal membrane oxygenation (VA ECMO) and a percutaneous transvalvular microaxial flow pump (Impella; Abiomed, Danvers, MA, USA), referred to as ECPELLA, has emerged as a promising therapeutic approach [1]. Although ECPELLA provides robust hemodynamic support, prolonged immobilization and mechanical ventilation often lead to respiratory complications, including atelectasis and ventilator-associated pneumonia (VAP) [2]. In addition, respiratory dysfunction during ECPELLA support may contribute to differential hypoxemia, which poses a significant risk of cerebral hypoxia [3,4]. Accordingly, it is important to preserve optimal pulmonary function during ECPELLA support. Prone positioning therapy has been well established as an effective intervention for respiratory dysfunction, particularly in acute respiratory distress syndrome (ARDS) [5,6]. Its advantages include improved dorsal alveolar recruitment, more homogeneous ventilation–perfusion matching, and reduced overdistension of ventral lung regions [7]. Studies have also shown its usefulness in hypoxemic patients with coronavirus disease 2019 and ARDS [8]. Moreover, prone positioning has been suggested to be effective and safe for managing hypoxemic respiratory failure in patients after cardiac surgery [9,10]. It may also help mitigate atelectasis and VAP, which are frequently observed in patients undergoing ECPELLA support. However, when applying prone positioning in standard cases of respiratory failure, the unique risk factors not typically encountered in this population (e.g., bleeding from cannulation sites and hemodynamic instability) must be carefully considered [11,12,13].

To date, there are limited data regarding the safety and efficacy of prone positioning therapy in patients with cardiogenic shock supported by ECPELLA [14,15]. Although prone positioning has been studied in patients with ARDS and those receiving ECMO support, its role in patients with cardiogenic shock supported by ECPELLA remains largely unexplored. ECPELLA represents a unique and complex clinical scenario involving dual mechanical circulatory support, with distinct physiological challenges such as differential hypoxemia and increased procedural risks related to multiple cannulation sites. Therefore, the feasibility, safety, and physiological impact of prone positioning in this population cannot be extrapolated from previous studies. This study primarily aimed to evaluate within-patient physiological changes in oxygenation and respiratory mechanics associated with prone positioning in patients with cardiogenic shock supported by ECPELLA. Comparisons between patients who did and did not undergo prone positioning were exploratory and descriptive in nature.

## 2. Materials and Methods

### 2.1. Study Design and Population

This was a single-center, retrospective, single-arm observational study including all consecutive patients who received ECPELLA support for cardiogenic shock between February 2020 and August 2024. Patients diagnosed with cardiogenic shock and subsequently treated with ECPELLA were included in this study. Cardiogenic shock was defined according to the Society for Cardiovascular Angiography and Interventions classification.

### 2.2. Inclusion Criteria

All consecutive patients with cardiogenic shock who received ECPELLA support during the study period and had available clinical and hemodynamic data were included.

### 2.3. Exclusion Criteria

The exclusion criterion regarding the absence of pre- and post-prone positioning measurements was applied specifically to the within-patient analysis of patients who underwent prone positioning. All patients receiving ECPELLA support were included in the overall cohort description.

Patients were excluded if they had (1) missing key clinical or respiratory data, (2) absence of pre- or post-prone positioning measurements, or (3) contraindications to prone positioning as judged by the attending physician.

### 2.4. Prone Positioning Cohort Definition

**Figure 1:**
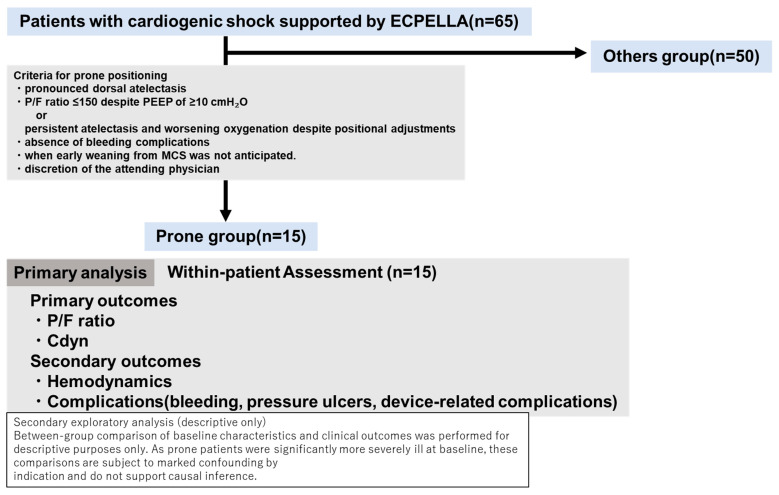
Study design, patient selection, and analytical framework. This figure illustrates the overall study design, including patient selection and analytical strategy. A total of 65 patients with cardiogenic shock supported by ECPELLA between February 2020 and August 2024 were retrospectively reviewed, of whom 15 underwent prone positioning for hypoxemic respiratory failure. The primary analysis focused on within-patient physiological changes associated with prone positioning, comparing respiratory parameters before and after the intervention. Secondary analyses included between-group comparisons (prone vs. non-prone positioning), which were conducted for descriptive purposes only due to baseline differences. The main outcomes assessed were oxygenation (P/F ratio), Cdyn, hemodynamic parameters, and procedure-related complications. Secondary outcomes included hemodynamic parameters and procedure-related complications. Abbreviations: P/F, PaO_2_/FiO_2_; PEEP, positive end-expiratory pressure; Cdyn, dynamic lung compliance.

Patients who underwent prone positioning were identified from among those who received ECPELLA support (Figure 1). Prone positioning was considered according to institutional practice in patients with pronounced dorsal atelectasis and a PaO_2_/FiO_2_ ratio ≤ 150 despite a positive end-expiratory pressure (PEEP) of ≥10 cmH_2_O, or in those with persistent atelectasis and worsening oxygenation despite positional adjustments. Prone positioning was performed at the discretion of the attending physician only in the absence of bleeding complications and when early weaning from mechanical circulatory support was not anticipated.

### 2.5. Clinical Parameters and Data Collection

To quantify the intensity of vasoactive medication using the maximum dose administered within a 24 h period, the vasoactive–inotropic score (VIS) was calculated as follows [16]:

VIS = dopamine (µg/kg/min) + dobutamine (µg/kg/min) + 100 × epinephrine (µg/kg/min) + 10 × milrinone (µg/kg/min) + 10,000 × vasopressin (units/kg/min) + 100 × norepinephrine (µg/kg/min)

The sequential organ failure assessment (SOFA) score was evaluated at admission to the intensive care unit. For patients with cardiac arrest, the Glasgow Coma Scale score was assigned as three points, and the SOFA neurological subscore was correspondingly set at four points.

To assess the effects of prone positioning on respiratory function and hemodynamics, the following data were collected: PaO_2_/FiO_2_ ratio (P/F ratio), dynamic lung compliance (Cdyn), and right heart catheterization parameters before and after prone positioning therapy.

To evaluate arterial oxygenation in the upper body, all arterial blood gas samples were obtained from the right radial artery. In patients supported with femoral VA-ECMO and Impella, this measurement reflects upper-body arterial oxygenation and may be influenced by ECMO flow, native cardiac output, ventilator settings, and the position of the mixing zone [17]. The mean arterial blood pressure (mABP) was used as a primary hemodynamic parameter because several patients in our cohort exhibited markedly reduced pulse pressure due to severe cardiac dysfunction. In such cases, systolic and diastolic blood pressure alone may not adequately reflect systemic perfusion status. Therefore, mABP was considered a more appropriate and reliable indicator of overall circulatory support. The mABP was calculated using the standard formula (diastolic blood pressure + 1/3 pulse pressure) and was obtained from invasive arterial pressure monitoring. Baseline (“before”) measurements were obtained within 1 h prior to initiation of prone positioning. “Immediately after” measurements were defined as those obtained 1 h after initiation of prone positioning. “After” measurements were obtained 1 h after return to the supine position following prone positioning. In patients who underwent multiple prone positioning sessions, only data from the first session were used for analysis to ensure consistency across patients. Adverse events were assessed across all prone positioning sessions.

### 2.6. Prone Positioning Therapy

Prone positioning was initiated in patients with hypoxemic respiratory failure, particularly when imaging revealed dorsal atelectasis, at the discretion of the attending physician. It was only performed when no contraindications (e.g., active bleeding or hemodynamic instability) were present. At our institution, a multidisciplinary team evaluated each patient for eligibility by confirming the absence of active bleeding (particularly at cannulation sites), ventricular arrhythmias (e.g., ventricular tachycardia or fibrillation), and frequent vomiting. During prone positioning, analgesia and sedation were managed to maintain a target Richmond Agitation–Sedation Scale (RASS) score of −5 to prevent cannulation site bleeding caused by body movement. Patient repositioning was performed by trained medical staff. PEEP was kept constant before and after repositioning. To maintain lung-protective ventilation, driving pressure was adjusted to keep tidal volume constant. Tidal volume was targeted at lung-protective levels (approximately 6 mL/kg predicted body weight), and ventilator settings were not systematically altered during the observation period. ECMO flow and Impella support levels were maintained according to clinical indications and were not systematically modified during prone positioning.

### 2.7. Outcome Measures

The primary outcomes of this study were changes in oxygenation and respiratory mechanics associated with prone positioning. Oxygenation was assessed using the P/F ratio, and respiratory mechanics were evaluated using Cdyn, both measured before and after prone positioning.

Secondary outcomes included hemodynamic changes associated with prone positioning, as assessed using parameters obtained from right heart catheterization, as well as the incidence of procedure-related complications. Complications of interest included bleeding events (particularly at cannulation sites), pressure ulcers, and device-related complications such as dislodgement of ECMO or Impella cannulas or endotracheal tubes. Between-group comparisons were performed for descriptive purposes only and were not intended to infer causal relationships.

### 2.8. Statistical Analysis

Statistical analyses were performed using JMP version 18. Continuous variables are presented as the median (interquartile range [IQR]). Meanwhile, categorical variables are presented as counts and percentages. Continuous variables were compared using the Mann–Whitney U test, whereas categorical variables were compared using Fisher’s exact test. Paired comparisons of the P/F ratio, lung compliance, and hemodynamic parameters before and after prone positioning were performed using the Wilcoxon signed-rank test. Statistical significance was considered at a two-tailed *p*-value < 0.05.

The study was approved by the institutional review board of Ichinomiya Municipal Hospital (Approval No. 1438-A2024092). Clinical trial registration was not required due to the study’s retrospective design. The requirement for informed consent was waived because of the study’s retrospective nature. An opt-out process was implemented to inform patients and provide an opportunity to decline participation. Generative AI tools were used only for language editing and improvement of manuscript readability. All scientific content, data interpretation, and conclusions were reviewed and verified by the authors.

## 3. Results

### 3.1. Study Population and Baseline Characteristics

A total of 65 patients received ECPELLA support for cardiogenic shock during the study period, of whom 15 underwent prone positioning for hypoxemic respiratory failure, primarily due to dorsal atelectasis. Among the 65 patients, VA ECMO was established via the femoral artery and femoral vein, and an Impella 2.5 or CP was inserted via the femoral artery in 64 patients. In the remaining patient, femoral access was not feasible due to descending aortic occlusion; therefore, VA ECMO was configured with venous drainage from the femoral vein and arterial return via the carotid artery, and an Impella 5.0 was inserted via the subclavian artery.

Baseline characteristics are summarized in Table 1 and Table S1. Compared with patients who did not undergo prone positioning (the others), those in the prone group had significantly higher SOFA scores and lower initial P/F ratios, indicating a more severe clinical status. Comparisons between the prone and the others should be interpreted as descriptive due to baseline differences.

### 3.2. Key Clinical Outcomes of Prone Positioning

Details of in-hospital management in the prone group are shown in Table 2. Half of the patients had acute myocardial infarction, and all underwent primary percutaneous coronary intervention (PCI). Two patients required ECPELLA support due to hemodynamic instability during staged PCI.

Prone positioning was initiated at a median of 2 days after ECPELLA initiation, mainly in response to hypoxemia attributed to dorsal atelectasis. Each session lasted a mean of 16 h, with a median cumulative duration of 32 h.

**Figure 2:**
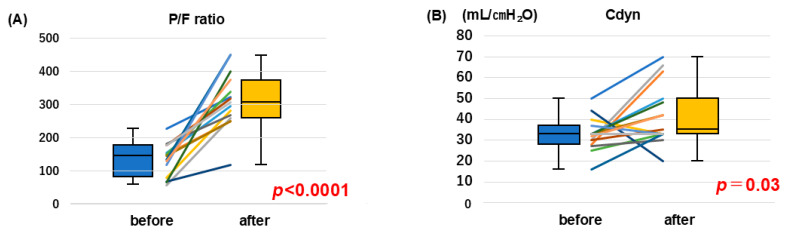
Comparison of P/F ratio (**A**) and Cdyn (**B**) before and after the prone position. Abbreviations: P/F, PaO_2_/FiO_2_; Cdyn, dynamic lung compliance; ECPELLA, VA ECMO combined with IMPELLA; IQR, interquartile range.

Prone positioning was associated with a marked improvement in respiratory parameters (Figure 2). The P/F ratio significantly increased from 145 (IQR, 80–177) to 308 (IQR, 260–375; *p* < 0.0001). Cdyn also showed a modest but statistically significant increase from 33 (IQR, 28–37) to 35 (IQR, 33–50) mL/cmH_2_O (*p* = 0.03).

### 3.3. Hemodynamic Stability

Hemodynamic parameters before, immediately after, and after prone positioning are presented in Table 3. No significant changes were observed in mABP, pulmonary artery pressures, cardiac index, heart rate, or vasoactive–inotropic score. The overall hemodynamic status remained stable during prone positioning.

### 3.4. Safety Outcomes and Complications

Adverse events related to prone positioning are summarized in Table 4. One patient (6.7%) experienced bleeding at the ECMO cannulation site during the third prone session, leading to early termination of the session. Hemostasis was successfully achieved with a purse-string suture after returning to the supine position, and no additional blood transfusion was required. No dislodgement of ECMO or Impella cannulae, nor endotracheal tube displacement, was observed. Pressure ulcers occurred in four patients (26.7%), all of which were classified as stage 1 or 2 according to the National Pressure Ulcer Advisory Panel classification. These were managed conservatively with moisturizing agents, gauze protection, or wound dressings, and no severe complications were observed.

### 3.5. Clinical Outcomes

Neurological outcomes and survival rates are presented in Table 5. A good neurological outcome was defined as CPC 1–2. The proportion of patients with a good neurological outcome at day 30 was 13.3% in the prone group and 36% in the others (*p* = 0.12). At day 90, the corresponding values were 20% and 44%, respectively (*p* = 0.13). Survival rates at day 30 were 60% in the prone group and 70% in the others (*p* = 0.54), and at day 90 were 33.3% and 56%, respectively (*p* = 0.15). Although the proportion of patients with good neurological outcome differed between the prone and the others, the difference was not statistically significant. Similarly, survival rates at both 30 and 90 days differed between groups but did not reach statistical significance. Given the baseline differences between groups and the potential for confounding by indication, these comparisons are presented for descriptive purposes only and should be interpreted with caution.

In 15 patients who underwent prone positioning during ECPELLA support, the changes before and after prone positioning were illustrated using line graphs and box-and-whisker plots. The box-and-whisker plots display the median (IQR), as well as the minimum and maximum values.

Prone positioning was associated with a marked improvement in respiratory parameters. The P/F ratio significantly increased from 145 (IQR, 80–177) to 308 (IQR, 260–375; *p* < 0.0001). Cdyn also showed a modest but statistically significant increase from 33 (IQR, 28–37) to 35 (IQR, 33–50) mL/cmH_2_O (*p* = 0.03).

## 4. Discussion

To the best of our knowledge, this study is one of the first studies to report the clinical application of prone positioning therapy in patients with cardiogenic shock supported by ECPELLA. Recent studies have increasingly evaluated the role of prone positioning in patients receiving ECMO, particularly in the context of severe ARDS. Several systematic reviews and meta-analyses have demonstrated that prone positioning during ECMO is associated with significant improvements in oxygenation and gas exchange, as reflected by increases in the P/F ratio and reductions in PaCO_2_ [18]. Furthermore, some recent meta-analyses suggest that prone positioning during ECMO may be associated with improved short-term survival, including reductions in 28-day and in-hospital mortality, although the certainty of evidence remains low and long-term survival benefits are less consistent [19]. Most of these studies have focused on patients receiving venovenous ECMO for ARDS, including COVID-19-related respiratory failure. In contrast, patients supported with ECPELLA represent a fundamentally different population characterized by the coexistence of severe cardiogenic shock and respiratory failure requiring dual mechanical circulatory support.

Importantly, the present study specifically focuses on patients receiving ECPELLA support, a population that differs substantially from those included in prior studies of prone positioning in ECMO or ARDS. This distinction highlights the novelty of our findings and their relevance to a previously underexplored clinical setting.

This retrospective analysis identified three key observations. First, the patients in the prone group were more critically ill compared with the others, non-prone, as indicated by the higher SOFA scores and lower initial P/F ratios. Second, prone positioning was associated with a marked improvement in oxygenation. In contrast, the increase in Cdyn was statistically significant but small and may not be clinically meaningful. Third, although several complications were observed, prone positioning appeared to be feasible, and the observed physiological improvements may outweigh the associated risks in this cohort.

### 4.1. Patient Severity in the Prone Group

Patients who received prone positioning therapy exhibited more severe illness at baseline. Compared with the others, patients who underwent prone positioning had higher SOFA scores and lower initial P/F ratios, indicating more pronounced systemic organ dysfunction and respiratory compromise. Moreover, the ECPELLA support duration was significantly longer in the prone group than the others (232 h vs. 123 h, *p* = 0.02), suggesting greater illness severity and a need for prolonged intensive care. Among patients who met the hypoxemia criteria for prone positioning but did not fulfill the criterion of marked atelectasis, eight patients were identified in the non-prone group. The baseline PaO_2_/FiO_2_ ratio in this subgroup was 120 (IQR, 80–137), which was not significantly different from that of the prone group (*p* = 0.19). In contrast, the SOFA score was 12 (IQR, 11–13), which was significantly lower in the non-prone subgroup compared with the prone group (*p* = 0.006).

Additionally, the ECPELLA support duration was 129 h (IQR, 76–176) in the others, but equally hypoxemic subgroup. Although this difference did not reach statistical significance, patients in this subgroup tended to be liberated from support earlier than those in the prone group. These findings suggest that, despite similar degrees of hypoxemia, patients who underwent prone positioning were overall more severely ill (Table S2).

Although the neurological outcomes were not significantly different, the proportion of patients with poor outcomes tended to be higher in the prone group than in the others (Table 5). This is attributed to the small sample size and the fact that prone positioning was selectively applied to patients already at high risk for adverse outcomes. Nonetheless, considering that the respiratory function improved after prone positioning, the outcomes in the prone group should be interpreted in the context of higher baseline severity.

The selective use of prone positioning in this population suggests that it was employed as a rescue strategy for refractory hypoxemia. This also highlights the clinical hesitancy to apply prone positioning in fragile patients with multiple risk factors. Nevertheless, our findings suggest that prone positioning may be a useful supportive strategy for respiratory management, even in high-risk patients.

### 4.2. Respiratory Function Improvement

Prone positioning was associated with significant improvements in respiratory parameters, including the P/F ratio and Cdyn. Although a statistically significant increase in Cdyn was observed after prone positioning, the absolute magnitude of this change was small and may not be clinically meaningful. This suggests that the primary physiological effect of prone positioning in this cohort was improvement in ventilation–perfusion matching rather than a substantial increase in lung recruitability. In contrast, the improvement in oxygenation was substantial. Importantly, in patients supported with femoral VA-ECMO and Impella, an improvement in the P/F ratio derived from right radial arterial blood cannot be interpreted as a direct improvement in native pulmonary oxygenation, but rather reflects an improvement in upper-body arterial oxygenation and differential hypoxemia.

The underlying physiological mechanisms are likely multifactorial. Prone positioning promotes dorsal lung recruitment, reduces atelectasis, and improves ventilation–perfusion matching. These are well-established mechanisms in ARDS.

In patients receiving ECPELLA support, these effects may be particularly relevant. During VA ECMO, oxygenated blood is delivered retrogradely from the femoral artery. In this setting, inadequate native lung oxygenation may result in differential hypoxemia, especially affecting the upper body and cerebral circulation. By improving native pulmonary oxygenation, prone positioning may shift the mixing zone proximally. This may enhance oxygen delivery to the brain and potentially reduce the risk of cerebral hypoxia. This mechanism may be especially important in ECPELLA-supported patients, in whom both systemic perfusion and pulmonary gas exchange are critically impaired. In addition, prone positioning may reduce regional lung overdistension. It may also improve right ventricular afterload by decreasing hypoxic pulmonary vasoconstriction. Although no significant changes in hemodynamic parameters were observed in our study, these physiological effects may contribute to the overall stability observed during prone positioning.

An important alternative to prone positioning in patients with inadequate oxygenation on VA ECMO is the addition of a venous reinfusion cannula to establish V-AV ECMO. This approach allows direct augmentation of oxygen delivery to both the arterial and venous circulations and can be performed at the bedside without the need for additional operating room time. Although V-AV ECMO is an effective strategy for improving oxygenation and facilitating lung-protective ventilation until pulmonary edema or acute respiratory distress syndrome improves, as well as for the correction of differential hypoxia, it does not directly address dorsal lung collapse. In patients with significant dorsal atelectasis, prone positioning can improve alveolar recruitment and ventilation–perfusion matching, and therefore represents a useful adjunctive strategy in this specific clinical setting. In the present study, prone positioning was primarily applied in patients in whom atelectasis was considered a major contributor to hypoxemia based on clinical and radiographic findings. However, we did not attempt to strictly differentiate between atelectasis-related hypoxemia and primary parenchymal lung disease, such as pneumonia or ARDS. Instead, our cohort reflects a real-world population in which multiple mechanisms of respiratory failure often coexist during ECPELLA support. These findings suggest a potential role for prone positioning as an adjunctive respiratory management strategy, although causal relationships cannot be established due to the retrospective single-arm design.

### 4.3. Safety and Complications of Prone Positioning

A previous study has observed various complications associated with prone positioning in mechanically supported patients, including bleeding, vomiting, catheter dislodgement, pressure ulcers, facial edema, nerve injury, endotracheal tube displacement, and airway obstruction [20].

Although complications remain a concern, our findings suggest that prone positioning can be safely implemented with appropriate precautions. Such complications can be mostly prevented or mitigated to avoid progression to severe outcomes with staff training and pressure relief using cushions, moisturizing pads, and wound dressings. No cases of ECMO or Impella cannula dislodgement or endotracheal tube displacement occurred. Although one patient (6.7%) experienced bleeding at the ECMO cannulation site during prone positioning (which necessitated the early termination of the session), the bleeding was promptly managed with a purse-string suture. Another patient developed a retroperitoneal hematoma before prone therapy was initiated; therefore, it was not considered to be related to prone positioning.

Bleeding is a well-recognized and significant complication associated with MCS. Previous studies have reported major bleeding events in approximately 30% of patients undergoing ECPELLA or Impella support [12,21]. In one study, bleeding at the cannulation site accounted for approximately 15% of cases [22]. In our study, the incidence of major bleeding did not differ significantly between the prone group and the others as assessed by the GUSTO criteria (Table S3). In the prone group, major bleeding occurred in 20% of patients, with cannulation site bleeding during prone therapy observed in 6.7%. These relatively low rates might be attributed to the routine use of purse-string sutures to secure the cannulation site. The incidence of pressure ulcers tended to be higher in the prone group than in the others, although the difference was not statistically significant (Table S3). In general, larger patients have a higher risk of pressure-related injuries, including pressure ulcers, during prone positioning and are therefore considered less suitable candidates for this intervention. In the present study, the largest patient who underwent prone positioning had a body surface area of 1.99 m^2^ and a body mass index of 29.2 kg/m^2^; however, no clinically apparent pressure ulcers were observed in this case. Given the small sample size, this study does not allow for a reliable assessment of the risk of prone positioning in this population. Further studies are required before its broader application can be recommended.

Prone positioning influences hemodynamics, with potential reductions in venous return. Conversely, it is associated with improvements in right ventricular function and overall hemodynamics through enhanced lung recruitment and reduced hypoxemia, hypercapnia, driving pressure, and plateau pressure [20]. In the present study, no significant hemodynamic changes were observed between the pre- and post-prone sessions (Table 3). The observed reduction in lactate was modest and may reflect improved systemic perfusion or reduced metabolic stress during prone positioning. However, no consistent changes were observed in standard hemodynamic variables, and causality cannot be established. These findings are consistent with previous reports suggesting that prone positioning can be safely performed in mechanically supported patients with appropriate precautions.

### 4.4. Clinical Implications for Patient Management

The present findings have several important clinical implications for the management of patients with cardiogenic shock, supported by ECPELLA.

First, prone positioning may be considered a feasible adjunctive strategy for improving oxygenation in patients with refractory hypoxemia, particularly when dorsal atelectasis is present. In such cases, it may be implemented before escalation to more invasive approaches, such as conversion to venoarterial–venous (V-AV) ECMO.

Second, our results suggest that prone positioning can be performed without significant hemodynamic compromise, even in patients receiving dual mechanical circulatory support. This may help alleviate concerns regarding circulatory instability and cannula-related complications, which often limit its use in this population.

Third, careful patient selection remains essential. In our cohort, prone positioning was primarily applied to patients with severe hypoxemia and radiographic evidence of dorsal atelectasis. These characteristics may help identify patients most likely to benefit from this intervention. Hypoxemia in patients receiving ECPELLA support is often multifactorial. Our findings suggest that prone positioning can be feasibly implemented even in such complex clinical conditions. It may therefore represent a practical adjunctive option for respiratory management. However, the physiological response may vary depending on the underlying pathology. Identifying patients who are most likely to benefit from this approach—particularly those with predominant atelectasis and refractory hypoxemia—remains an important area for future research.

Finally, prone positioning should be performed within a multidisciplinary framework, with attention to cannula stabilization, bleeding risk, and pressure injury prevention. Standardized protocols and staff training may further enhance the safety and feasibility of this approach.

Taken together, these findings suggest that prone positioning may represent a practical and relatively low-risk adjunctive strategy for respiratory management in selected patients with cardiogenic shock receiving ECPELLA support. However, further prospective studies are needed to define its optimal indications and timing.

### 4.5. Limitations

This study has several limitations that should be acknowledged.

First, it was a retrospective, single-center study with a relatively small sample size, which limited the statistical power. Therefore, multivariate analysis was not performed to avoid model overfitting. Accordingly, the findings should be interpreted as exploratory and hypothesis-generating.

Second, the study lacked a standardized protocol for prone positioning. The decision to initiate prone positioning was made at the discretion of the attending physician based on clinical judgment, which may have introduced selection bias. In particular, patients in the prone group had more severe baseline conditions, as reflected by higher SOFA scores and lower P/F ratios, suggesting that prone positioning was preferentially applied to more critically ill patients.

Third, the absence of a matched control group limits the ability to draw causal inferences. Comparisons between the prone and the others should therefore be interpreted as descriptive rather than causal, and the observed associations may be confounded by unmeasured variables.

Fourth, several potential confounders were not fully accounted for, including differences in ventilator management, sedation strategies, fluid balance, and timing of intervention, all of which may have influenced respiratory and hemodynamic outcomes. Detailed quantitative support settings were not consistently available, which may limit the precise interpretation of oxygenation changes.

Fifth, the evaluation of outcomes was limited primarily to short-term physiological changes and in-hospital outcomes. Long-term outcomes, including functional recovery and quality of life, were not assessed.

Finally, the relatively small number of patients undergoing prone positioning limits the ability to adequately assess the safety profile, particularly for rare but clinically significant complications such as cannula dislodgement or major bleeding.

Therefore, the findings of this study should be considered hypothesis-generating, and further prospective, multicenter studies with standardized protocols are warranted to validate these results.

## 5. Conclusions

In this single-center, retrospective study, prone positioning was feasible in selected patients with cardiogenic shock supported by ECPELLA and was associated with improved oxygenation.

No significant hemodynamic deterioration was observed during prone positioning, and complications were manageable within the study cohort. However, given the study design, small sample size, and potential selection bias, these findings should be interpreted with caution and do not establish causality. Prone positioning may be considered a supportive option for managing hypoxemia in carefully selected patients, but further prospective, multicenter studies are required to clarify its efficacy, safety, and optimal indications.

## Figures and Tables

**Table 1:** Characteristics of the study subjects.

	All, *n* = 65	Prone, *n* = 15	The Others, *n* = 50	*p*
Age, years	66 (56–74)	73 (65–76)	64 (54–73)	0.01
Male, *n*	53 (82%)	13 (86%)	40 (80%)	0.71
BSA, m^2^	1.69 (1.57–1.84)	1.73 (1.58–1.83)	1.68 (1.56–1.85)	0.49
BMI, kg/m^2^	22 (21–25)	22 (21–26)	22 (20–25)	0.30
Etiology, *n*				
AMI	38 (58%)	8 (53%)	30 (60%)	0.77
Cardiac myocarditis	5 (8%)	2 (13%)	3 (6%)	0.33
Cardiomyopathies	17 (26%)	3 (20%)	14 (32%)	0.74
Other	5 (8%)	2 (13%)	3 (6%)	0.33
VIS	11 (2–21)	11 (0–21)	11 (2–25)	0.68
SOFA	13 (11–15)	15 (14–16)	12 (11–14)	<0.0001
P/F ratio	180 (140–305)	145 (80–177)	230 (168–320)	<0.0001

Categorical variables are described as counts and percentages, and continuous variables are described using median and interquartile range. Patients who underwent prone positioning were compared with those who did not (the others). Abbreviations: BSA, body surface area; BMI, body mass index; AMI, acute myocardial infarction; VIS, vasoactive–inotropic score; SOFA, sequential organ failure assessment; P/F, PaO_2_/FiO_2_.

**Table 2:** Details of treatment and clinical course of the study subjects undergoing prone positioning during ECPELLA.

	Prone, *n* = 15
MCS time, hours	232 (103–259)
The average number of the prone, times	2 (1–3)
The total duration of the prone, hours	32 (21–48)
CRRT due to oliguria, *n*	13 (86.7%)
Total transfusion volume (RBC), units	20 (16–30)
Complication of atelectasis, *n*	15 (100%)

Categorical variables are described as counts and percentages, and continuous variables are described using median and interquartile range. Abbreviations: MCS, mechanical circulatory support; CRRT, continuous renal replacement therapy; RBC, red blood cell.

**Table 3:** Comparison of hemodynamic parameters before and after the prone position and immediately after initiating the prone position.

	Before	Immediately	After	*p*
mABP, mmHg	83 (72–91)	81 (75–91)	81 (73–93)	0.33
sPAP, mmHg	32 (22–37)	29 (22–37)	30 (25–35)	0.25
dPAP, mmHg	21 (16–25)	20 (15–22)	20 (16–21)	0.08
PAPP, mmHg	10 (5–15)	11 (6–15)	10 (7–16)	0.77
CVP, mmHg	13 (9–17)	15 (9–17)	13 (9–16)	0.29
CI, L/min/m^2^	1.5 (1.1–2.3)	1.3 (0.9–1.8)	1.7 (1.3–2.3)	0.12
HR, beats/min	76 (63–86)	77 (66–90)	82 (64–91)	0.25
lactate, mmol/L	1.6 (1.3–1.9)	1.5 (1.4–1.8)	1.3 (1.2–1.5)	0.02
VIS	10.1 (0–16.0)	10.1 (0–17.7)	10.7 (0–12.4)	0.65

Continuous variables are described as median and interquartile range. Statistical comparisons were performed only between “before” and “after” measurements using the Wilcoxon signed-rank test. The “immediately after” values are shown for descriptive purposes only and were not included in statistical testing. *p*-values are presented for descriptive interpretation, and no adjustment for multiple comparisons was applied. Abbreviations: mABP, mean arterial blood pressure; sPAP, systolic pulmonary artery pressure; dPAP, diastolic pulmonary artery pressure; PAPP, pulmonary artery pulse pressure; CVP, central venous pressure; CI, cardiac index; HR, heart rate; VIS, vasoactive–inotropic score.

**Table 4:** Adverse events observed during prone positioning.

Bleeding at the Insertion Site, *n*	1 (6.7%)
dislodgement of the ECMO or Impella cannulae, *n*	0 (0%)
Endotracheal tube displacement or obstruction, *n*	0 (0%)
Pressure ulcer(degree), *n*	4 (26.7%)
I	2 (13.3%)
II	2 (13.3%)
III	0 (0%)
IV	0 (0%)

Categorical variables are described as counts and percentages. Abbreviations: ECMO, extracorporeal membrane oxygenation; Impella, percutaneous transvalvular microaxial flow pump.

**Table 5:** Comparison of neurological outcomes between the prone positioning group and the others during ECPELLA.

	Prone, *n* = 15	The Others, *n* = 50	*p*
CPC (day 30)			0.55
1	2 (13.3%)	15 (30.0%)	
2	0 (0%)	3 (6.0%)	
3	2 (13.3%)	7 (14.0%)	
4	5 (33.3%)	10 (20.0%)	
5	6 (40.0%)	15 (30.0%)	
CPC (day 90)			0.46
1	3 (20.0%)	17 (34.0%)	
2	0 (0%)	5 (10.0%)	
3	1 (6.7%)	2 (4.0%)	
4	1 (6.7%)	4 (8.0%)	
5	10 (66.7%)	22 (44.0%)	

Categorical variables are described as counts and percentages. Abbreviations: CPC, cerebral performance categories.

## Data Availability

The datasets used and/or analyzed during the current study are available from the corresponding author on reasonable request.

## Supplementary Materials

The following supporting information can be downloaded at: https://www.mdpi.com/article/10.3390/jcm15103626/s1, Table S1: Comparison of characteristics between the prone positioning group and the others; Table S2: Comparison of characteristics between the prone positioning group and the hypoxemic subgroup without prone positioning; Table S3: Comparison of adverse events between the prone positioning group and the others during ECPELLA.

## Abbreviations

The following abbreviations are used in this manuscript:

MCS	mechanical circulatory support
VA ECMO	venoarterial extracorporeal membrane oxygenation
Impella	percutaneous transvalvular micro-axial flow pump
ECPELLA	VA ECMO combined with IMPELLA
VAP	ventilator-associated pneumonia
ARDS	acute respiratory distress syndrome
PEEP	positive end-expiratory pressure
VIS	vasoactive–inotropic score
SOFA	sequential organ failure assessment
P/F	PaO_2_/FiO_2_
Cdyn	dynamic lung compliance
mABP	mean arterial blood pressure
RASS	Richmond Agitation–Sedation Scale
IQR	interquartile range
PCI	percutaneous coronary intervention
