# Supplementary material for: Prone Positioning During ECPELLA Support for Cardiogenic Shock: A Single-Center Retrospective Study
Source: J Clin Med. 2026 May 9;15(10):3626. doi: 10.3390/jcm15103626 (PMC13206796; doi:10.3390/jcm15103626)
Supplement: Supplementary file 1 [file jcm-15-03626-s001.zip › jcm-4217694-supplementary.pdf]

・**Table S1 Comparison of characteristics between the prone positioning group and the others**

	All, N=65	Prone, N=15	The others, N=50	P
Comorbidities				
Hypertension, n	33(51%)	9(60%)	24(48%)	0.56
Diabetes, n	23(35%)	8(53%)	15(30%)	0.13
Dyslipidemia, n	25(38%)	9(60%)	16(32%)	0.07
Renal failure, n	32(49%)	7(47%)	25(50%)	1.00
Pulmonary disease, n	4(6%)	1(7%)	3(6%)	1.00

Categorical variables are described as percentages.
Pulmonary disease: COPD(chronic obstructive pulmonary disease) or IP(interstitial pneumonia)

・Table S2 Comparison of characteristics between the prone positioning group and the hypoxemic subgroup without prone positioning

	Prone, N=15	the hypoxemia in the others, N=8	P
Age, years	73(65-76)	59(47-71)	0.04
Male, n	13(86%)	7(87%)	1.00
BSA, m^2	1.73(1.58-1.83)	1.88(1.67-2.00)	0.08
BMI, kg/m^2	22(21-26)	24(22-28)	0.67
Etiology, n			
AMI	8(53%)	5(62%)	1.00
Cardiac myocarditis	2(13%)	0(0%)	0.52
Cardiomyopathies	3(20%)	3(37%)	0.62
Other	2(13%)	0(0%)	0.52
VIS	11(0-21)	2(0-25)	0.79
SOFA	15(14-16)	12(11-13)	0.006
P/F ratio	145(80-177)	120(80-137)	0.19
MCS support time, hours	232(103-259)	129(76-176)	0.12
Comorbidities			
Hypertension, n	9(60%)	6(75%)	0.65
Diabetes, n	8(53%)	3(37%)	0.66
Dyslipidemia, n	9(60%)	3(37%)	0.40
Renal failure, n	7(47%)	6(75%)	0.15
Pulmonary disease, n	1(7%)	1(12%)	1.00

Categorical variables are described as percentages and continuous variables using median and interquartile range. Patients who underwent prone positioning were compared with the hypoxemic subgroup without prone positioning.
Abbreviations: BSA; body surface area, BMI; body mass index, AMI; acute myocardial infarction, VIS; vasoactive-inotropic score, SOFA;
Sequential Organ Failure Assessment, P/F; PaO2/FiO2, MCS; mechanical circulatory support

・Table S3 Comparison of adverse events between the prone positioning group and the others during ECPELLA

	Prone, N=15	The others, N=50	P
Severe bleeding(GUSTO criteria)	3(20.0%)	7(14.0%)	0.69
Pressure ulcer(degree), n	4(26.6%)	10(20.0%)	0.66
Ⅰ	2(13.3%)	3(6.0%)	
Ⅱ	2(13.3%)	7(14.0%)	
Ⅲ	0(0%)	0(0%)	
Ⅳ	0(0%)	0(0%)	

Categorical variables are described as percentages.
